# Generation of secondary microbial methane of high-rank coals: insights from the microbial community and carbon isotope

**DOI:** 10.3389/fmicb.2024.1414379

**Published:** 2024-08-01

**Authors:** Hui Nai, Sheng Xu, Biying Chen, Jun Zhong, Lujia Fang, Sirou Qin, Yuji Sano

**Affiliations:** ^1^Institute of Surface-Earth System Science, School of Earth System Science, Tianjin University, Tianjin, China; ^2^Marine Core Research Institute, Kochi University, Kochi, Japan

**Keywords:** coalbeds, microbial community, δ^13^C, secondary microbial methane, high-rank coal

## Abstract

Secondary microbial methane could provide a valuable energy source if it were better understood. Although coal seam is an ideal environment for investigating secondary microbial methane, there are few studies to trace the secondary microbial methane of high-rank coals. Here, we collected co-produced water samples from coalbeds in the Qinshui Basin (China) and analyzed the microbial community structure by 16S ribosomal RNA (16S rRNA) amplicon sequencing analysis. 16S rRNA sequencing demonstrated abundant methanogens in coalbeds including 6 orders (Methanobacteriales, Methanococcales, Methanofastidiosales, Methanomassiliicoccale, Methanomicrobiales, and Methanosarciniales) and 22 genera of methanogens. Superheavy DIC (δ^13^C_DIC_ ranging from −4.2‰ to 34.8‰) and abundance of methanogenic microbes in co-produced water revealed the generation of secondary biogenic methane in high-rank coal seams in the Qingshui Basin. Hydrogenotrophic methanogenesis is the main pathway for secondary biogenic methane production. In deeply buried coal seams, biogenic methane is dominated by CO_2_ and H_2_ reduction methanogenesis, and in shallow buried coal seams, it may be produced synergistically by hydrocarbon degradation and hydrogenotrophic methanogenic microbes. The study discussed here is important for a better understanding of the generation of secondary microbial methane in high-rank coal.

## 1 Introduction

In recent years, with the voracious demand and depletion of conventional energy sources, there is an urgent demand for the development of unconventional energy sources (Mohr et al., 2015; Tutak and Brodny, 2022). Coal bed methane (CBM) is increasingly becoming an important source of energy around the world, such as in the United States, Australia, China, India, and Canada (Moore, 2012). The CBM mainly originates from biogenic or thermogenic processes (Scott, 1993; Scott et al., 1994; Whiticar, 1999; Moore, 2012). Biogenic CBM accounts for 30% of global CBM resources (Fallgren et al., 2013) and is possibly active during the entire coal formation period (Moore, 2012). Given the large contribution of biogenic methane in CBM systems, studying the microbial formation process of CBM has important theoretical and practical significance for understanding the generation mechanism and increasing the production of CBM.

Primary biogenic CBM generates at the early stages of coal formation and generally dissipates during prolonged crustal movements (Wang A. K. et al., 2018), making secondary biogenic methane crucial for CBM resources (Faiz and Hendry, 2006; Wang A. K. et al., 2018). The breaking down of complex organic compounds to methanogenic substrates in coal is considered the rate-limiting step in secondary biogenic methane formation (Strapoc et al., 2011). Heteroatoms including oxygen, sulfur, and nitrogen in coal are lost during the coalification, resulting in the formation of more recalcitrant compounds that are challenging for microbial breakdown (Levine, 1993; Xiao et al., 2013). Hence, low-maturity coals are believed to have greater potential for secondary biogenic gas production while high-rank coals are less likely to host biogenic methane (Robbins et al., 2016; Sepulveda-Castaneda et al., 2022; Tang et al., 2022; Chen et al., 2023). However, recent studies have shown that high-rank coal (e.g., bituminous coals) also has the ability to produce biogenic methane (Haider and Rahim, 2017; Malik et al., 2020; Chen et al., 2024b), and this potential may be higher than that of lower-rank coal seams (Fallgren et al., 2013). Widespread and active bacteria and archaea in coal reservoirs play a vital role in the formation of secondary biogenic CBM through synergistic degradation of organic matter (Strapoc et al., 2011; Colosimo et al., 2016; Vick et al., 2018). The understanding of the generation processes of secondary biogenic gas in high-rank coal seams is crucial for the evaluation of natural gas resources and for enhancing the production of biogenic methane via microbial stimulation (Chen et al., 2024b). However, limited knowledge exists about the exact origins of secondary biogenic CBM associated with microbial communities in high-rank coalbeds.

The Qinshui Basin is one of the largest basins of high-rank coals in China. In this study, we collected co-produced water samples from six regions in the northern, central, and southern Qinshui Basin with a wide range of vitrinite reflectance (Ro). We investigated the in-situ generation processes of secondary biogenic methane in the high-rank coalbeds, by analyzing microbial community compositions of coal co-produced water together with other geochemical proxies such as the stable carbon isotope of dissolved inorganic carbon (δ^13^C-DIC). We hypothesize that secondary biogenic methane formation exits in high-rank coal seam. This research contributes significantly to a deeper understanding of the biogenic processes in CBM formation and holds crucial value for the development and utilization of CBM resources.

## 2 Geological setting

**Figure 1:**
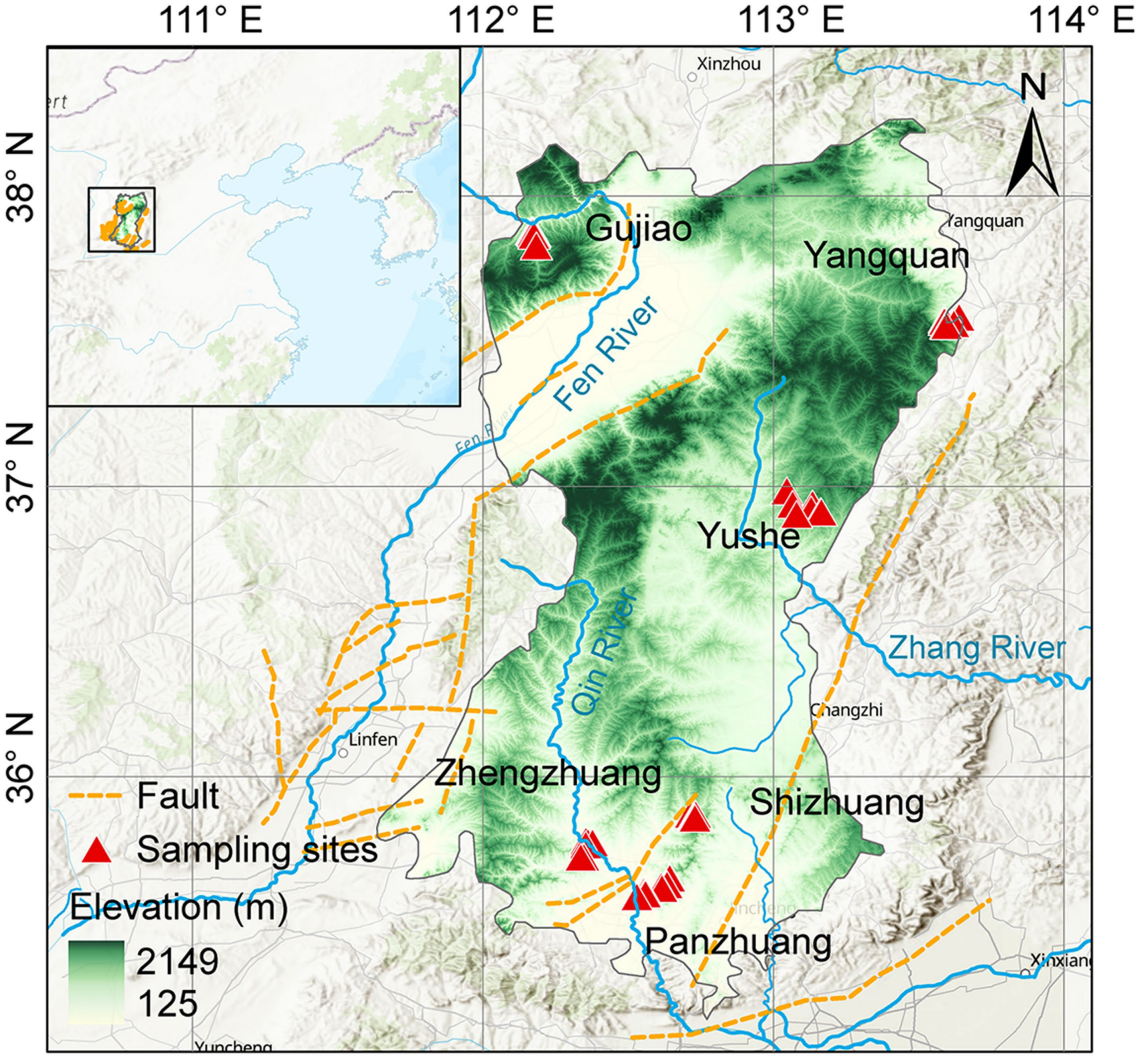
Location of the Qinshui Basin and distribution of CBM co-produced water sampling wells.

The Qinshui Basin is located in the north-central part of China (Figure 1), with an area of ~23,923 km^2^. It is one of the most productive CBM basins in China (Zhao and Shi, 2005). In terms of tectonic location, the Qinshui Basin is situated in the central part of the North China Craton Basin, between the uplifts of the Taihang Mountains, Zhongtiao Mountains, Huoshan Mountain, and Wutai Mountain, forming a large-scale complex syncline trending in a north-northeast direction (Liu et al., 2022). The basin has been in an uplifted state since the Late Jurassic (Zhao et al., 2005), with developed internal folding and relatively underdeveloped faulting. The main structural faults in the region are the Sitou Fault in the south and the Jinhuo Fault in the east, in addition to several minor faults within the region.

The lithology of the Qinshui Basin is primarily composed of Paleozoic-Mesozoic sedimentary rocks (Song et al., 2018). The upper Carboniferous Taiyuan Formation and the lower Permian Shanxi Formation are the main coal-bearing strata in the basin, with an average total thickness of ~150 m and more than 10 coal seams (Teng et al., 2015) and a total coal thickness of about 1.2–23.6 m (Su et al., 2005; Song et al., 2018). The Taiyuan Formation and the Shanxi Formation are widely distributed throughout the region. The thickness of the main coal seam of the Shanxi Formation is about 0.5–7.8 m, with a greater thickness in the southeastern of the basin (Lu et al., 2019). The burial depth overall is shallow in the northeast-east-southeast and deep in the central part, ranging from 200 to 3,000 m (Liu et al., 2022). The buried depth of the main coal seam of the Taiyuan Formation is overall 100 m deeper than that of the Shanxi Formation (Jiang et al., 2023). The thickness of its main coal seam is about 0.6–9.9 m, showing a general trend of increasing thickness from north to south (Zhao et al., 2005; Liu et al., 2022).

The Qinshui Basin transformed into a marine–terrestrial transitional coal-bearing basin in the Carboniferous-Permian period (Liu et al., 2022). Influenced by multiple tectonic movements and the Yanshan period magmatic–metamorphic activity (Su et al., 2005; Cao et al., 2015), the coal seams are mainly anthracite rank (Su et al., 2005). The Ro of the coal seam is generally >2.5%, and the highest Ro can reach 4.5% (Jiang et al., 2023). Overall, the maturity of the coal seams shows a pattern of higher maturity in the south and lower in the north. The Panzhuang region (PZ) in the southern part has a Ro exceeding 4%, the Zhengzhuang region (ZZ) has a Ro of 2.8–4%, and the Shizhuang region (SZ) has a Ro of about 2.4% (Zhang et al., 2018; Liu et al., 2022). The Ro in the Yushe region (YS) and Yangquan region (YQ) are ~2.3% and ~ 3.1%, respectively (Xu et al., 2016; Xie et al., 2022). The maturity of the coal in the Gujiao region (GJ) in the northwest is the lowest, with Ro of 1.2–2.0% (Li et al., 2018; Wang Y. et al., 2018). The coal seams mainly experienced a late Permian to late Triassic thermal-gas generation stage and a late Yanshan concealed magmatic intrusion-induced thermal secondary gas generation stage, with mainly thermogenic coalbed gas (Zhao et al., 2005).

The major aquifers in the Qinshui Basin include the Ordovician limestone confined aquifer, the Taiyuan limestone-sandstone confined aquifer, the Shanxi -Shihezi sandstone confined aquifer, and the Quaternary sand-gravel phreatic aquifer (Zhang et al., 2017). The aquitards mainly consist of mudstone at the bottom of the coal-bearing strata, the underlying Ordovician Fengfeng Formation, and the hundred-meter-thick interbedded sand-mudstone between the Taiyuan Formation and the Shanxi Formation, with little hydraulic connection between the layers (Zhang et al., 2015, 2017). Consistent with the North China region, the hydrodynamic conditions of the lower Taiyuan Formation coal measures in the Qinshui Basin are stronger than those of the upper Shanxi Formation.

## 3 Materials and methods

### 3.1 Sample collection and processing

Field surveys and sample collections were conducted in July 2022 and February 2023 from the Qinshui Basin including GJ, YQ, YS, SZ, PZ, and ZZ regions (Figure 1). A total of 27 CBM co-produced water samples were collected through water extraction pipe for CBM wellheads and 2 river water samples were collected from Qin River. Electrical conductivity (EC) and pH were measured immediately after sampling using a multi-parameter water quality analyzer (3,630 IDS, WTW, Germany), and the total alkalinity of the water sample was titrated in situ using 0.02 M HCl, and the DIC concentration was further calculated based on the ion balance using Phreeqc software (v3.6.2, USGS) (Fang et al., 2024). For each sample, 125 mL water was filtered through a 0.22 μm filter (MEC, Millipore), and sealed in polyethylene bottles for the measurements of δ^13^C-DIC which was analyzed using a GasBench device interfaced with a Delta V stable isotope mass spectrometer (Thermo Fisher, United States) (Fang et al., 2024). (Since DIC and CO_2_ in co-produced water have the same origins and there is a relatively stable fractionation (8‰) between their ^13^C, δ^13^C-DIC and δ^13^C-CO_2_ are interchangeable to characterize inorganic carbon isotope variations throughout the manuscript). For the determination of the microbial community in this study, 2 L water samples were collected into sterile water bags for high-throughput sequencing.

### 3.2 DNA extraction and PCR amplification of 16S rRNA genes

27 of the CBM co-produced water samples and 2 of the river water samples for 16S rRNA genes analysis were conducted in this study. ArBa515F (5′-GTGCCAGCMGCCGCGGTAA-3′) and Arch806R (5′-GGACTACVSGGGTATCTAAT-3′) targeted the V4 region of the 16S rRNA gene was used for the amplification of bacteria and archaea (Bates et al., 2011). DNA extraction, PCR amplification, and gene sequencing were determined according to Cui et al. (2018). The overlapped paired-end sequences obtained through Illumina Miseq platform were assembled to tags (FLASH v1.2.11) and quality filtered with the QIIME (v1.9.1) pipeline. Clustering was performed using the UPARSE (v11) to obtain operational taxonomic units (OTUs) at a 97% similarity threshold. Taxonomic annotation was further performed using the RDP classifier (v2.13) with a 0.7 confidence threshold as cutoff. To quantify the abundance of the 16S rRNA, the Real-time PCR assay was performed through an ABI 7300 sequence detection system (Applied Biosystems, United States). The 16S rRNA genes sequencing and quantification were analyzed at Majorbio Bio-Pharm Technology Co., Ltd. (Shanghai, China). The microbial functional annotation was further inferred based on the microbial taxonomy using the FAPROTAX database (v1.2.7), which is powerful for disentangling biogeochemical cycling functions from the taxonomic community (Louca et al., 2016).

## 4 Results

### 4.1 Hydrochemistry of CBM co-produced water

Table 1 lists some hydrochemical parameters of CBM co-produced water from the six regions in the Qinshui Basin. The pH of the CBM co-produced water varies from 7.5 to 9.1 with an average value of 8.2 ± 0.4, indicating neutral to alkalescent water. The EC is in the range of 1,032 to 19,830 μS cm^−1^ with an average value of 4,134 ± 4,515 μS cm^−1^. In comparison to the GJ, YQ, SZ, and PZ regions, the YS and ZZ regions exhibit lower pH and higher EC. Coalbed methane extraction injects KCl-added local river water into the wells. Zhang et al. (2016) proposed a [Cl] > 10 mM as a boundary to distinguish the presence of fracturing fluid in the CBM co-produced water. Little influence of fracturing fluid could be discovered in GJ (median Cl concentration, 3.4 mM, same as below), YQ (1.2 mM), SZ (3.9 mM), and PZ (3.5 mM) regions, but there is still some residual in YS (20.6 mM) and ZZ (23.5 mM) regions (Fang et al., 2024). In addition, the CBM wells in the YS and ZZ regions are deeper having well depths all exceeding 1,450 m, compared to GJ, YQ, SZ, and PZ regions (316–824 m).

**Table 1:** Water chemistry and diversity indexes of the microbial community of CBM co-produced water.

Well ID	Block	EC (μS cm^−1^)	pH	DIC (mM)	δ^13^C_DIC_ (‰)	Well depth (m)	Copies (×10^9^ L^−1^)	Ace	Chao	Coverage	Shannon	Simpson
XST153	GJ	1,403	8.5	10.5	27.1	328	0.031	370.6	367.8	0.998	3.13	0.10
XST144	GJ	1,032	8.7	7.3	7.6	316	0.099	336.2	341.8	0.997	2.28	0.19
XST123	GJ	1,495	7.9	10.9	30.6	489	0.14	567.8	551.1	0.996	2.61	0.17
XSD086	GJ	5,940	8.1	22.8	30.1	521	0.67	602.3	575.0	0.995	2.61	0.22
YQ01	YQ	1,361	9.1	12.5	22.6	385	0.38	291.5	282.9	0.998	2.36	0.19
YQ15	YQ	1,569	8.7	16.1	19.7	713	0.16	492.7	467.3	0.996	2.38	0.22
YQ255	YQ	1,554	8.7	16.0	19.4	652	0.11	384.0	337.4	0.997	2.35	0.15
YQ329	YQ	1,504	8.8	15.3	22.9	569	0.63	281.9	240.7	0.998	1.82	0.25
ZK1202	YS	9,090	7.9	24.1	12.6	1,579	1.95	366.1	313.8	0.998	2.04	0.26
ZK121	YS	6,700	7.7	14.1	34.8	1,458	2.37	294.3	281.5	0.998	1.81	0.43
ZK1212	YS	19,830	8.1	18.9	32.9	1,491	4.43	231.0	219.8	0.998	2.01	0.20
ZK0714	YS	17,100	7.9	14.4	20.0	1,518	0.89	370.0	351.5	0.997	2.71	0.12
ZK1302	YS	4,110	7.7	27.7	29.2	1,514	0.53	290.1	273.0	0.998	2.64	0.19
TS007	SZ	1,575	7.9	10.6	12.3	716	0.0016	378.6	327.3	0.997	2.15	0.26
TS680	SZ	1743	8.7	17.8	31.7	824	0.72	507.0	442.4	0.997	2.68	0.19
TS675	SZ	5,410	8.7	68.2	−4.2	772	5.64	518.6	530.1	0.997	3.23	0.10
TS672	SZ	1,225	8.3	11.2	11.6	740	1.04	519.4	509.4	0.996	2.90	0.11
ZHL711	ZZ	4,110	7.9	25.6	1.2	1946	5.26	269.7	213.1	0.998	1.03	0.66
ZHL71	ZZ	5,280	8.0	23.1	21.6	1738	13.7	273.5	220.7	0.998	1.01	0.60
CZBL03	ZZ	3,270	7.5	25.1	2.8	1956	3.38	451.4	393.5	0.997	2.04	0.32
ZHL251	ZZ	3,640	7.9	23.9	0.8	1813	15.1	372.5	294.2	0.997	1.63	0.37
ZHL471	ZZ	2,810	7.9	24.2	8.3	1,564	7.16	481.7	428.9	0.997	2.51	0.18
CZ104	PZ	2,200	8.0	11.8	5.9	568	0.50	158.0	159.6	0.999	1.30	0.44
CZ0871	PZ	2030	8.5	10.6	7.3	560	6.81	556.4	535.8	0.996	1.95	0.40
SHCK163	PZ	2,410	7.5	2.8	−2.6	524	0.072	606.8	608.7	0.996	3.98	0.04
CZ315	PZ	1,403	8.8	15.2	4.4	421	9.18	158.7	160.6	0.999	2.21	0.17
SHU28V	PZ	1956	8.7	16.2	15.6	442	1.28	988.4	849.5	0.993	3.34	0.08
HHHS	River	780	8.3	4.0	−10.9	–	7.07	1282.7	915.9	0.992	3.54	0.07
QHPZ	River	503	8.5	2.6	−8.7	–	7.01	1976.2	1670.9	0.985	4.19	0.05

Data of pH, EC, DIC concentration, and δ^13^C-DIC were from Fang et al. (2024). ZZ, YS, SZ, PZ, GJ, and YQ correspond to Zhengzhuang, Yushe, Shizhuang, Panzhuang, Gujiao, and Yangquan, respectively.

The average DIC concentration in CBM co-produced water is 18.4 ± 11.6 mM. The DIC content in the ZZ and YS regions is significantly higher than in other regions (Table 1, Kruskal-Wallis test, *p* < 0.01). In addition, TS675 from the SZ region exhibits the highest DIC concentration in all CBM co-produced water, which could be attributed to the injection of CO_2_ (Zhou et al., 2013; Chen et al., 2024b).

The δ^13^C-DIC of river water varies a narrow range from −10.9‰ to −8.7‰ (Fang et al., 2024), which is consistent with the δ^13^C-DIC of global river water (Atekwana et al., 2003; Doctor et al., 2008; Dubois et al., 2010; Cai et al., 2015; Chen et al., 2021). The δ^13^C-DIC of CBM co-produced water varies in a wide range from −4.2‰ to 34.8‰, which is much heavier than that of global groundwater samples (average − 14.9‰) (Jasechko, 2019) but similar with the δ^13^C-DIC of CBM co-produced water in other coal reservoirs worldwide (McIntosh et al., 2010; Sharma et al., 2014; Yang et al., 2020).

**Figure 2:**
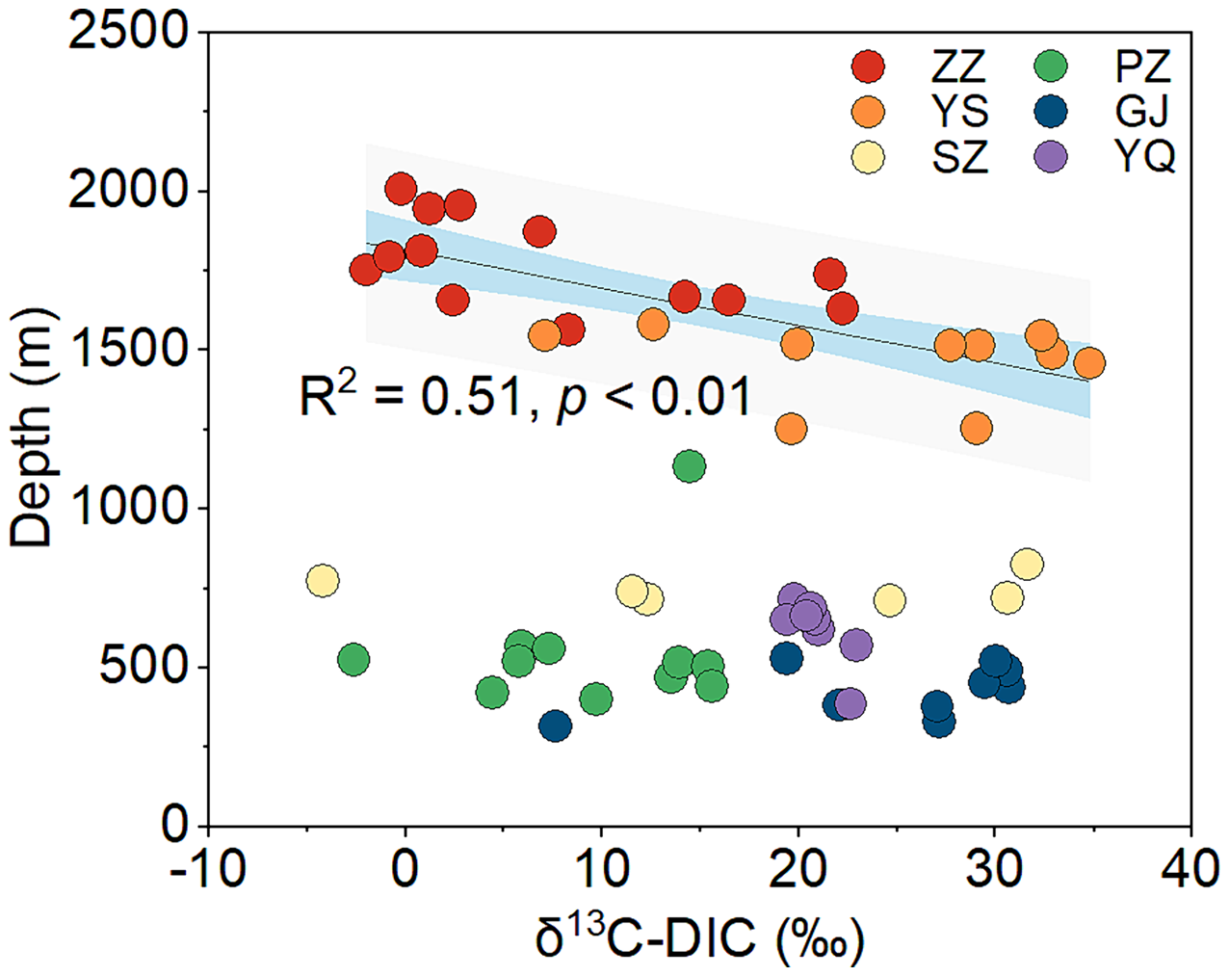
The correlation between δ^13^C-DIC and coalbed well depth in the six regions from Qinshui Basin. ZZ, YS, SZ, PZ, GJ, and YQ correspond to Zhengzhuang, Yushe, Shizhuang, Panzhuang, Gujiao, and Yangquan, respectively.

The Kruskal-Wallis test revealed significant differences in δ^13^C-DIC of CBM co-produced water among the six regions in the Qinshui Basin (*p* < 0.01). Among the six regions, YS region (median, 28.4‰) has the heaviest δ^13^C-DIC, followed by GJ (27.1‰), YQ (20.7‰), SZ (18.5‰), and PZ (9.7‰), while ZZ region (2.8‰) has the lightest δ^13^C-DIC. Although there was a significant difference (*p* < 0.01) in δ^13^C-DIC between YS and ZZ regions, the δ^13^C-DIC in these two regions were significantly negatively correlated with coalbed well depth, whereas this correlation was not observed in the other regions with shallow well depth (Figure 2).

### 4.2 16S rRNA gene abundance

The 16S rRNA gene abundance in the two river water samples is very similar, with HHHS and QHPZ being 7.1 × 10^9^ copies L^−1^ and 7.0 × 10^9^ copies L^−1^, respectively (Table 1). However, the abundance of 16S rRNA genes in CBM co-produced water exhibits a wide range of variation, ranging from 1.6 × 10^6^ copies L^−1^ to 1.5 × 10^10^ copies L^−1^ (Table 1). Among the six regions, the ZZ region in the southern part of the basin has the highest gene abundance (8.92 ± 4.65 × 10^9^ copies L^−1^), followed by the PZ region (3.57 ± 3.71 × 10^9^ copies L^−1^) in the southern region, YS (2.03 ± 1.37 × 10^9^ copies L^−1^) region in the central region, and then the SZ region (1.85 ± 2.22 × 10^9^ copies L^−1^) in the southern region. The YQ (3.2 ± 2.1 × 10^8^ copies L^−1^) and GJ (2.3 ± 2.6 × 10^8^ copies L^−1^) regions in the northern region have the lowest number of gene copies.

### 4.3 Microbial community

All water samples in this study yielded 5,939,578 original sequences with sequences in each sample ranging from 35,108 to 133,418. After subsampling each sample to the minimum number of sequences (30,149 sequences pre-sample), filtering out sequences aligned to chloroplasts and mitochondria, and clustering at a 97% similarity threshold, a total of 4,201 OTUs were obtained, with the number of OTUs per sample ranging from 116 to 1,090. The number of OTUs in river water samples ranged from 537 to 1,090, while in CBM co-produced water samples it ranged from 116 to 534. The coverage index of the samples ranged from 0.985 to 0.999 (Table 1), indicating that the sequencing results represented a near-complete sampling of the microbial community (Roswell et al., 2021).

Community richness and diversity vary with regions (Table 1). The Chao1 and Ace indexes are commonly used to estimate the total number of species in ecological studies, with higher values indicating higher species richness (Chao, 1984; Chao and Lee, 1992). The Shannon and Simpson indexes are used to evaluate species diversity, with a higher Shannon or a lower Simpson index indicating stronger biodiversity (Guiasu and Guiasu, 2003). The microbial richness and diversity in river water were much higher compared to CMB co-produced water. Overall, there were also differences in microbial richness and diversity among the six regions. Compared to the GJ, SZ, and PZ regions, the YS, ZZ, and YQ regions exhibited lower species richness and diversity (Table 1).

**Figure 3:**
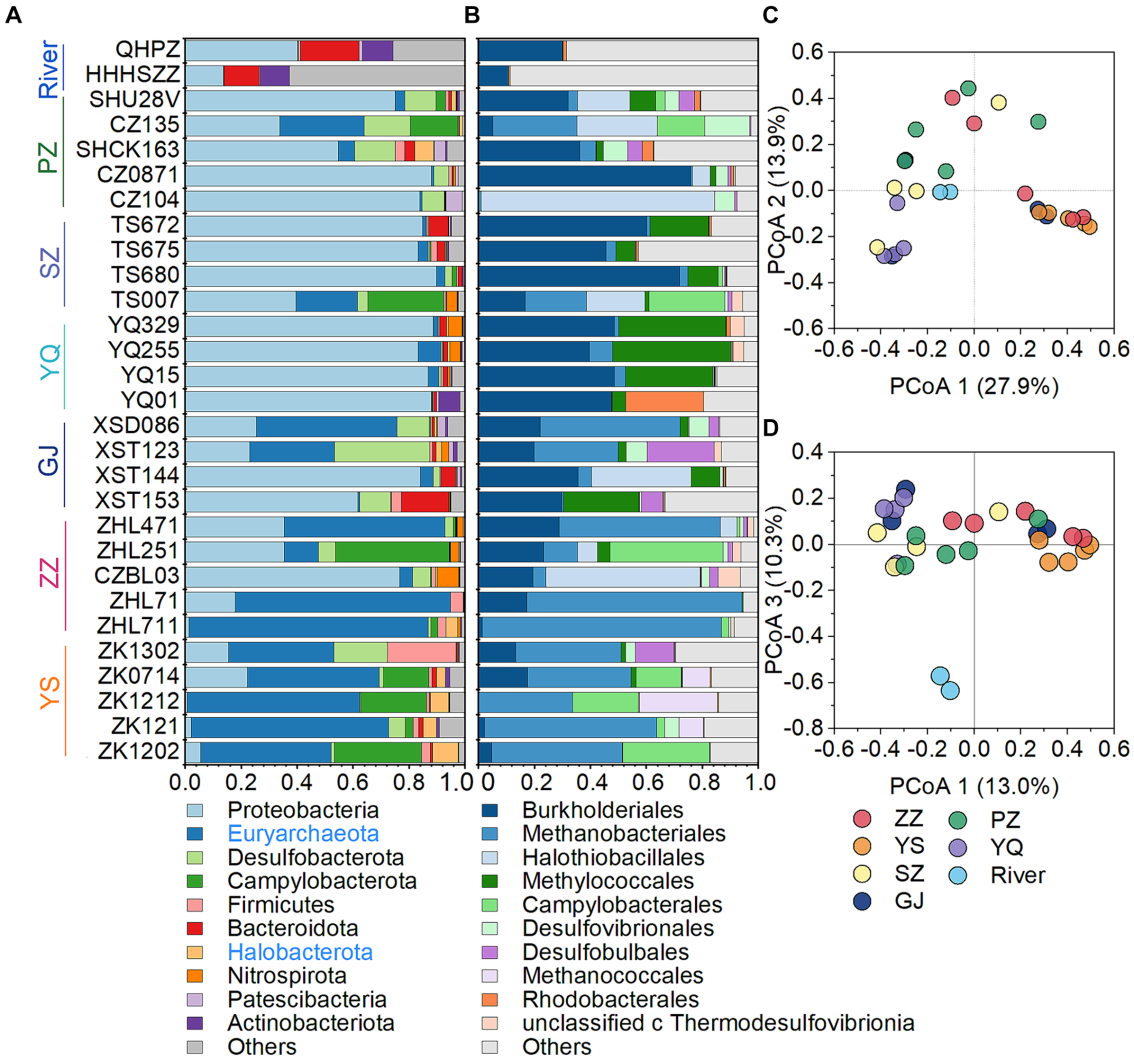
The microbial community structure of the CBM co-produced water and river water at the phylum level **(A)**, at the order level **(B)**, and principal co-ordinates analysis at the OTU level **(C,D)**. The archaeal phylum is presented in blue in the legend. ZZ, YS, SZ, PZ, GJ, and YQ correspond to Zhengzhuang, Yushe, Shizhuang, Panzhuang, Gujiao, and Yangquan, respectively.

The microbial community in CBM co-produced water was composed of 26.5% archaea and 73.5% bacteria. Overall, the most abundant phyla in CBM co-produced water were Proteobacteria (51.3%), Euryarchaeota (24.8%), Desulfobacterota (6.3%), Campylobacterota (6.2%), Firmicutes (2.2%), Bacteroidota (1.9%), Halobacterota (1.6%), Nitrospirota (1.2%), Patescibacteria (0.7%) and Actinobacteria (0.7%) (Figure 3A). Among the first ten phyla, Euryarchaeota and Halobacterota are archaea, which include many cultured or putative methanogens (Evans et al., 2019; Martijn et al., 2020). The relative abundance of two phyla of archaea, particularly Euryarchaeota, was higher in the YS and ZZ regions compared to the GJ, YQ, SZ, and PZ regions (Figure 3A). At the order level, most of the microbe were affiliated with Burkholderiales (21.7%), Methanobacteriales (21.5%), Halothiobacillales (9.0%), Methylococcales (7.6%), Campylobacterales (5.8%), as well as Desulfovibrionales (2.7%), Desulfobulbales (2.4%), Methanococcales (1.6%), Rhodobacterales (1.5%) and unclassified_c_Thermodesulfovibrionia (1.1%) (Figure 3B). The principal co-ordinates analysis (PCoA) at the OTU level declared that the microbial community structure was distinct between river waters and CBM co-produced water (Figure 3D). The microbial compositions of the YS and ZZ regions were also different from the other four regions (Figure 3C).

## 5 Discussion

A lot of geochemical proxies have been used to identify secondary microbial methane (i.e., McIntosh et al., 2010; Sharma et al., 2014; Yang et al., 2020). In particular, the δ^13^C-DIC has been considered to be an effective tracer for secondary microbial methane in CBM (i.e., Sharma et al., 2014). Isotopically superheavy δ^13^C-DIC (16.5 ± 10.7‰) was observed in CBM co-produced water of Qinshui Basin where high-rank coal was dominated. In general, superheavy carbon isotope of CO_2_ or DIC in coalbeds is usually recognized in low to medium-rank coal (Yang et al., 2020; Sepulveda-Castaneda et al., 2022; Tang et al., 2022; Wang et al., 2022), and rarely in high-rank coal (Malik et al., 2020). The source of DIC in CBM co-produced water is consistent with that of CO_2_ in coalbed gas, which might originate from atmospheric CO_2_, soil CO_2_, thermal decomposition of kerogen, hydrocarbon oxidation, carbonate dissolution, and mantle degassing (Shuai et al., 2013). Among these, only carbonate dissolution could generate positive δ^13^C-CO_2_, but it is typically <5‰ (Feyzullayev and Movsumova, 2010). Although fractionation can be caused by strong CO_2_ degassing, this has previously been reported to lead δ^13^C-DIC values of less than 13‰ (Zhang et al., 1995; Evans et al., 2008). In addition, the multiple sets of aquifers overlying the coalbed in the Qinshui Basin are expected to hinder the escape of gases (Li et al., 2016). However, the δ^13^C-DIC value in the CBM co-produced water in the Qinshui Basin ranged from −4.2‰ to 34.8‰ with 83% of samples over 5‰ and 63% of samples over 13‰ (Table 1). Therefore, the heavy carbon isotope signature is not expected to be from mixing of DIC from different sources or from CO_2_ degassing.

Superheavy DIC or CO_2_ is also recognized to occur in biologically active coal seams (Boreham et al., 1998; Barker and Dallegge, 2006; Li et al., 2016; Owen et al., 2016), as microorganisms exhibit a preference for utilizing ^12^C in microbial methane generation leading to ^13^C enrichment in residual products (e.g., DIC) (Etiope et al., 2009; Feyzullayev and Movsumova, 2010). In this way, microbial reactions involving CO_2_ are likely responsible for the formation of superheavy δ^13^C-DIC in CBM co-produced water of the Qinshui Basin. The Qinshui Basin coals are of high thermal maturity due to the structure thermal maturation events mainly during the late Triassic-Early Jurassic and Yanshan Stage (Ren et al., 2005; Zhao et al., 2005). During the thermal evolution process, the indigenous microorganisms in the coal seams were sterilized due to high temperatures. After the Yanshan Stage, the palaeogeo-temperature continued to decrease (Ren et al., 2005), which gradually favored the survival of microorganisms. Since the Cenozoic, the Qinshui Basin has experienced rapid basin inversion and stratigraphic uplift (Zhao et al., 2005). Moreover, the Himalayan tectonic movement led to the extensive development of local fissures inside the basin (Zhao et al., 2005), which provided favorable pathways for an influx of meteoric water with microbial communities (Strapoc et al., 2011; Li et al., 2016). Chen et al. (2024a) declared that the co-produced water in the Qinshui Basin is a mixture of old formation water (0.13 to 5 Ma) and a small amount of modern water (< 70 years). This evidence suggests the coal reservoirs in the Qinshui Basin may have experienced microbe reinoculation from the meteoric water since the Cenozoic, thus simulating the process of secondary biogenic methanogenesis within the coal bed system. Meanwhile, continuous aquifers in the Qinshui Basin provide an enclosed system for the production and storage of CBM. The CBM mining process injected fracking fluids in the last two decades, which is river water artificially mixed with KCl. The high concentration of KCl (~2%) is detrimental to the survival of microorganisms (Ma et al., 2020). In addition, PCoA analysis indicates that the microbial community in the river water differed from that in the co-produced water (Figure 3D), suggesting that the microorganisms were unlikely introduced by the injection of artificial fracturing fluids. Two distinct patterns of δ^13^C-DIC in CBM co-produced water were observed in the Qinshui Basin (Figure 2). There is a significant negative correlation between δ^13^C-DIC and coalbed well depth in the YS and ZZ regions, but this relationship does not exist in the GJ, YQ, SZ, and PZ regions. Simultaneously, beta-diversity demonstrates that the microbial community also exhibits differences between shallow and deep coal seams (Figures 3C,D). This suggests differences in the pathways of microbial formation between deep and shallow coals, resulting in different patterns of heavy δ^13^C-DIC.

**Figure 4:**
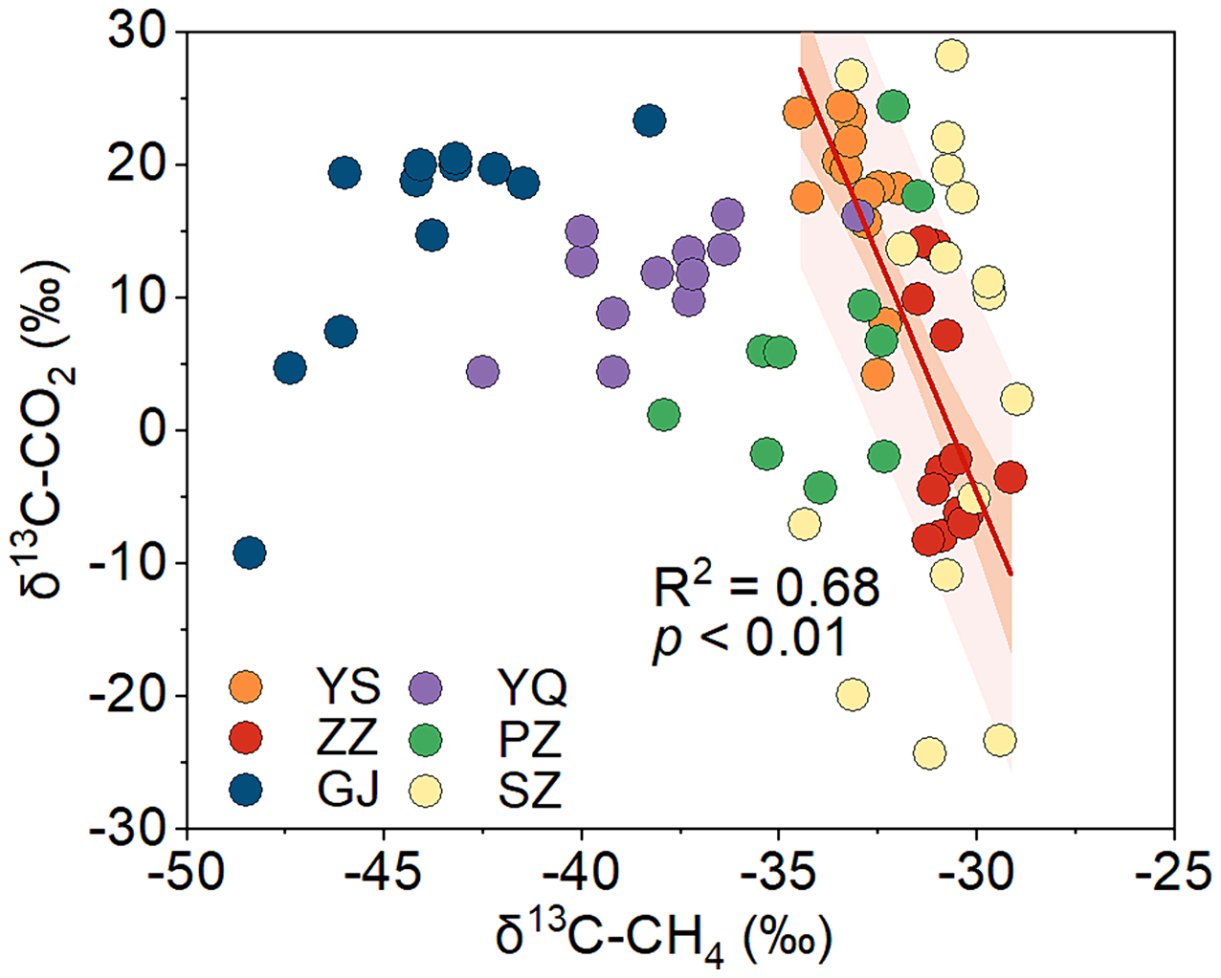
The relationship between δ^13^C-CH_4_ and δ^13^C-CO_2_ of coalbed gas in Qinshui Basin (data from Chen et al., 2024b). The line denotes the best fitting resulted from data in the ZZ and YS blocks. ZZ, YS, SZ, PZ, GJ, and YQ correspond to Zhengzhuang, Yushe, Shizhuang, Panzhuang, Gujiao, and Yangquan, respectively.

Biogenic methane is mainly produced through the CO_2_ reduction pathway and the acetate fermentation pathway in coal (Strapoc et al., 2011). The carbon isotope of the methyl in natural organic matter is lighter than that of the carboxyl (Su et al., 2023). In the acetate fermentation pathway, acetate is biologically decomposed into CO_2_ and CH_4_, during which methane is synthesized from methyl dehydrogenation and CO_2_ from carboxyl dehydrogenation (Gelwicks et al., 1994). This results in a lower value of δ^13^C-CH_4_ and a higher value of δ^13^C-CO_2_. In CO_2_ reduction methanogenesis, microbial enzymatic reactions will also lead to the ^12^C-enrichment in CH_4_ and the ^13^C-enrichment in residual CO_2_ (Conrad, 2005). Both the methanogenic pathways of CO_2_ reduction and acetate fermentation would cause an inverse change in δ^13^C-CH_4_ and δ^13^C-CO_2_/δ^13^C-DIC. According to the stable carbon isotopes of CO_2_ and CH_4_ in coalbed gas in the Qinshui Basin (Chen et al., 2024b), a significant negative correlation was found between the δ^13^C-CH_4_ and δ^13^C-CO_2_ in the ZZ and YS regions (Figure 4), indicating the occurrence of biogenic methane in these two regions.

**Figure 5:**
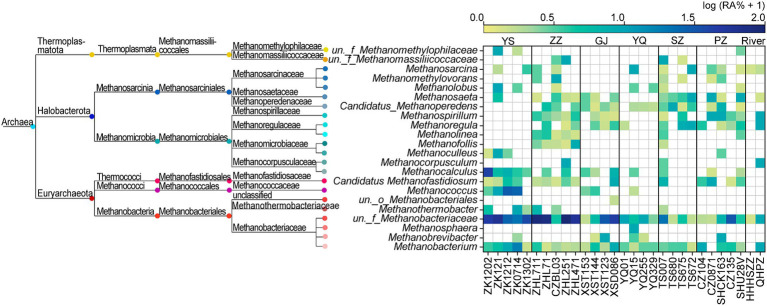
Phylogenetic tree of methanogens and the relative abundance (RA, %) heatmap at genus-level in the Qinshui Basin. ZZ, YS, SZ, PZ, GJ, and YQ correspond to Zhengzhuang, Yushe, Shizhuang, Panzhuang, Gujiao, and Yangquan, respectively.

**Figure 6:**
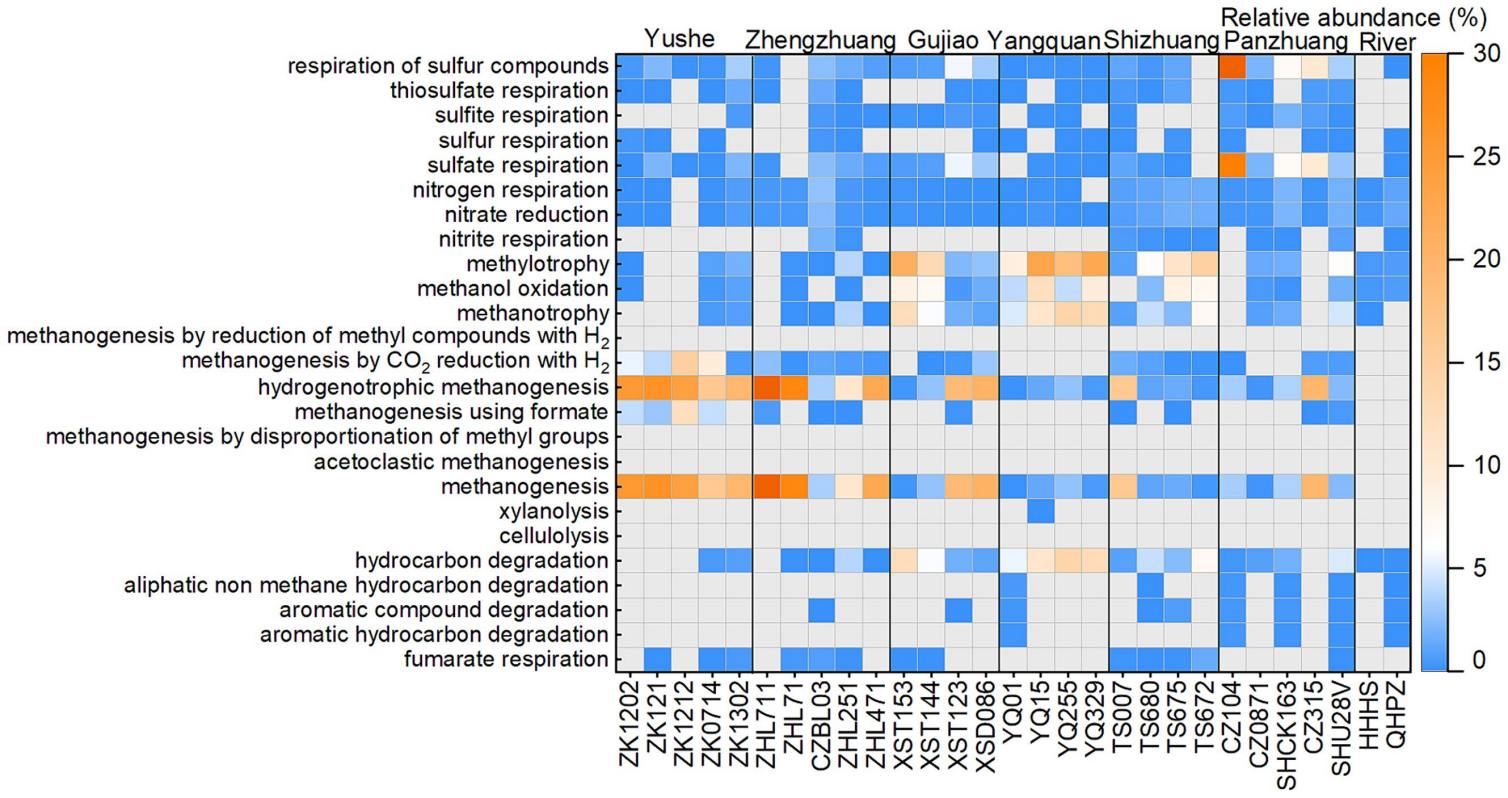
The heatmap of microbial functions predicted from FAPROTAX database.

**Figure 7:**
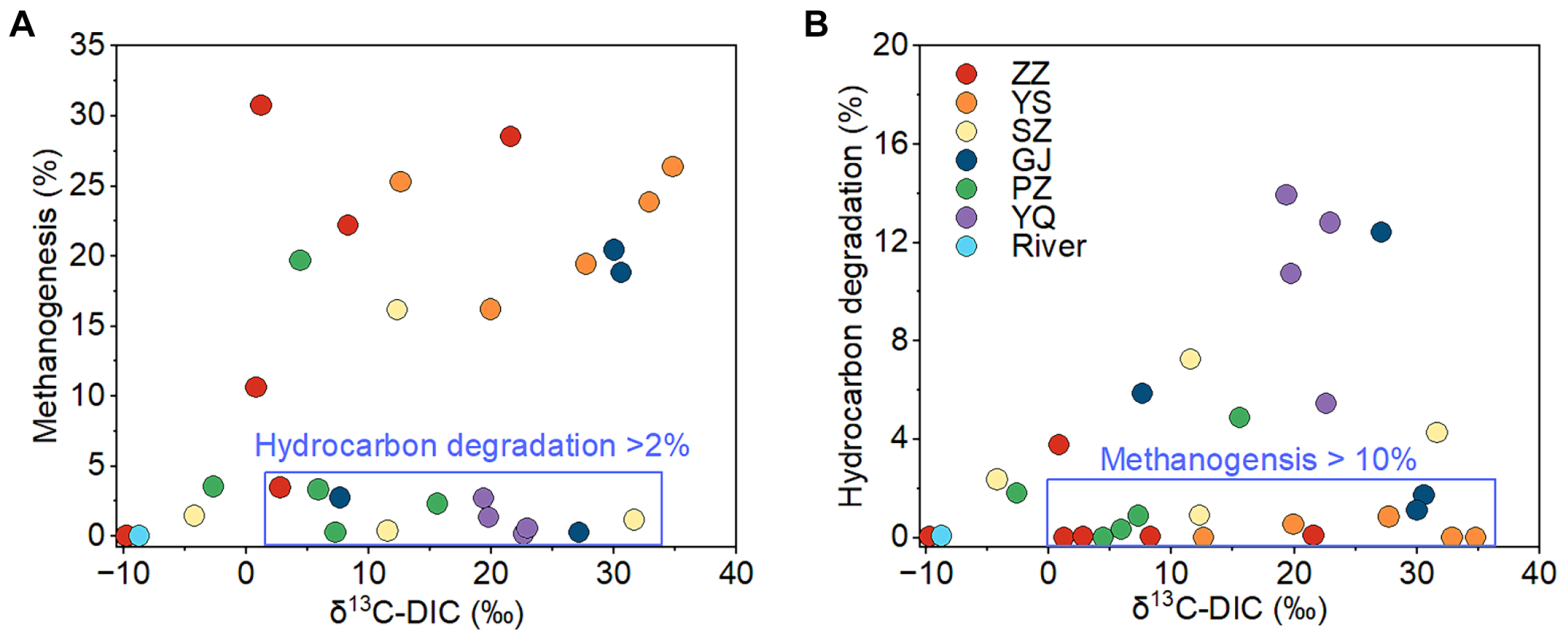
The relationship between δ^13^C-DIC and relative abundance of methanogens **(A)** and between δ^13^C-DIC and relative abundance of hydrocarbon degradation microbes **(B)** of CBM co-produced water in Qinshui Basin. ZZ, YS, SZ, PZ, GJ, and YQ correspond to Zhengzhuang, Yushe, Shizhuang, Panzhuang, Gujiao, and Yangquan, respectively.

The ZZ and YS regions with deeper well depths exhibit a higher relative abundance of archaea (Figure 3A). Thus far, eight orders of methanogens have been cultured, including the Methanococcales, Methanopyrales, Methanobacteriales, Methanomicrobiales, Methanocellales, Methanonatronarchaeales, Methanosarchinales, and Methanomassillicoccales (Bräuer et al., 2020). In the Qinshui Basin, Methanobacteriales, Methanococcales, Methanofastidiosales, Methanomassiliicoccale, Methanomicrobiales, and Methanosarciniales were found in the CBM co-produced water, and these orders could be further classified taxonomically into 14 families and 22 genera (Figure 5). The order Methanobacteriales, Methanococcales, Methanofastidiosales, and Methanomicrobiales exhibited a high relative abundance in the ZZ and YS regions, far exceeding that in the other four regions (Figures 3B, 5), which includes mainly methanogens that grow with CO_2_ reduction (Angelidaki et al., 2011). Although *Methanosarcina* and *Methanosaeta*, which have the potential of acetoclastic methanogenesis were detected, their relative abundance was relatively low (Figure 5). In this way, the contribution of acetoclastic methanogenesis to biogenic methane in the Qingshui Basin may be limited. Furthermore, based on the microbial functions of the CBM co-produced water calculated by the FAPROTAX database (Louca et al., 2016), a high relative abundance of methanogens, particularly methanogens previously found capable of CO_2_ reduction, was observed in the YS and ZZ regions (Figure 6). Indeed, as shown in Figure 7A, there is a considerable positive correlation between δ^13^C-DIC and the relative abundance of methanogenesis (>5%). These pieces of evidence suggest that secondary microbial methane generated by CO_2_ reduction is occurring in deeper buried high-maturity in the Qinshui Basin.

The process of CO_2_ reduction to CH_4_ seems insufficient in explaining the ^13^C-enriched DIC in the GJ, YQ, SZ, and PZ regions, as there is no significantly negative correlation between δ^13^C-CH_4_ and δ^13^C-CO_2_ (Figure 4) and there is low relative abundance of CO_2_-reducing methanogens (Figure 6). In contrast to the YS and ZZ regions, which had a high relative abundance of archaea (52.9% ± 26.1%), the bacteria predominated in the GJ, YQ, SZ, and PZ regions (89.1% ± 14.2%). The pervasive presence of bacteria, particularly Proteobacteria, Firmicutes, and Actinobacteria in these four regions (Figure 3A) favors the degradation of hydrocarbons in coalbed (An et al., 2016; Li et al., 2023). Previous studies suggested that the isotopically superheavy CO_2_ could be synthesized by the biodegradation of petroleum hydrocarbons (Pallasser, 2000). In anaerobic environments, hydrocarbons could be degraded by microorganisms to produce CO_2_ and further generate CH_4_ with the synergy of methanogenic consortia, where CO_2_ serves as an important intermediate product in this process and the δ^13^C-CO_2_ could reach around 25.9‰ (Jiménez et al., 2012; Xiao et al., 2013; Lu et al., 2022). This trend may be observed here, as there is a considerable positive correlation between δ^13^C-DIC and the relative abundance of hydrocarbon degradation (>2%) (Figure 7B) in the Qinshui Basin, which is based on the microbial function indicated by the FAPROTAX database.

The relative abundance of hydrocarbon-degrading bacteria predicted by FAPROTAX may be underestimated since the database is based on taxa affiliated with functional groups that mostly have been cultured (Louca et al., 2016). According to the methanogenic hydrocarbon-degrading consortia summarized by Jiménez et al. (2016), diverse hydrocarbon degradation bacteria was discovered in the Qinshui Basin, including Chloroflexi, Firmicutes, Thermotogae, Desulfobacterales, Desulfovibrionales, Rhizobiales, Geobacteraceae, Nocardiaceae, Anaerolineaceae, Nitrospiraceae, *Hydrogenophaga*, *Hyphomonas*, *Mycobacterium*, *Rhodococcus*, *Desulfobulbus*, *Desulfomonile*, *Desulfuromonas*, *Legionella*, *Marinobacter*, *Pseudomonas*, *Sphingomonas*, *Smithella*, *Syntrophus*, *Syntrophobacter*, and *Syntrophorhabdus*. These hydrocarbon-degrading bacteria have a higher relative abundance (22.1% ± 16.3%) in the GJ, YQ, SZ, and PZ regions compared to the YS and ZZ regions (9.8% ± 9.1%). Meanwhile, hydrogenotrophic methanogenesis was also discovered in the co-produced water in the GJ, YQ, SZ, and PZ regions (Figures 5, 6). These microbial communities support the transformation of hydrocarbons to methane in the electron acceptor-depleted environment (Jiménez et al., 2016; Ross et al., 2022). The hydrogen in this process might originate from groundwater and/or be a product of hydrocarbon degradation (Strapoc et al., 2011; Liu et al., 2020). In addition, Xiao et al. (2013) revealed that volatile materials could be degraded to bio-methane by methanogenic consortia in coal reservoirs in the Qinshui Basin. Chen et al. (2024b) found that the Qinshui Basin’s YS and ZZ regions had more C_2+_ gases than the GJ, YQ, and PZ regions, which perhaps indicates the occurrence of hydrocarbon consumption. This evidence suggested that the superheavy DIC in CBM co-produced water from the GJ, YQ, SZ, and PZ regions may suggest hydrocarbon degradation synergizing with hydrogenotrophic methanogens as another pathway of biological methane production in the shallow coalbed in the Qinshui Basin.

Thus, our results of the microbial community together with δ^13^C-DIC demonstrate that there are two pathways to generate secondary microbial methane of high-rank coals in Qinshui Basin: CO_2_-reducing methanogens in deep coalbed regions and hydrocarbon degradation in shallow coalbed regions. The question arises as to what kind of potential factors might influence these pathways at varying depths within the basin. The presence of two pathways for secondary microbial methane generation in high-rank coals within the Qinshui Basin is likely influenced by a combination of depth-dependent environmental conditions (i.e., temperature, pressure, availability of CO_2_ and O_2_, presence of hydrocarbon), organic carbon availability (i.e., maturity of coal deposits and microbial activity), and geological factors (i.e., coal composition and age, fluid flow dynamics, etc.) operating at different depths within the basin. These factors collectively contribute to the distinct microbial processes observed at different depths. Further investigations are required to clarify these trends in the future.

## 6 Conclusion

This study investigated the microbial compositions together with δ^13^C-DIC of CBM co-produced waters from high-rank coal seams in the Qinshui Basin. The composition of the microbial community showed that coal seam water is rich in methanogens, including 6 orders (Methanobacteriales, Methanococcales, Methanofastidiosales, Methanomassiliicoccale, Methanomicrobiales, and Methanosarciniales) and 22 genera of methanogens. The prevalence of superheavy DIC and methanogens in coalbed water in the Qinshui Basin suggests that secondary biogenic methanogenesis is occurring in high-maturity coal seams. The pathways of biogenic methane formation are depth-dependent. The CO_2_ and H_2_ reduction methanogenesis pathway predominates in deeply buried coal seams; biogenic methane in shallowly buried coal seams is functionally acted upon by hydrocarbon-degrading bacteria and hydrogenotrophic methanogenic microbes. Our findings provide insights for understanding the formation process of biogenic gas in high-rank coalbeds and might carry significant implications for enhancing coalbed methane.

## Data availability statement

The data presented in the study are deposited in the NCBI repository, accession number PRJNA1096746.

## Author contributions

HN: Writing – review & editing, Writing – original draft, Methodology, Investigation. SX: Writing – review & editing, Project administration, Funding acquisition. BC: Writing – review & editing, Investigation. JZ: Writing – review & editing, Conceptualization. LF: Writing – review & editing, Methodology, Investigation. SQ: Writing – review & editing, Methodology. YS: Writing – review & editing.
